# Oxidative stabilization of polyacrylonitrile nanofibers and carbon nanofibers containing graphene oxide (GO): a spectroscopic and electrochemical study

**DOI:** 10.3762/bjnano.8.161

**Published:** 2017-08-07

**Authors:** İlknur Gergin, Ezgi Ismar, A Sezai Sarac

**Affiliations:** 1 Polymer Science and Technology, Istanbul Technical University, Maslak, 34469 Istanbul, Turkey; 2 Nanoscience and Nanoengineering, Istanbul Technical University, Maslak, 34469 Istanbul, Turkey; 3 Department of Chemistry, Istanbul Technical University, Maslak, 34469 Istanbul, Turkey

**Keywords:** carbon nanofiber, graphene oxide, oxidized polyacrylonitrile (PAN)

## Abstract

In this study, a precursor for carbon nanofibers (CNF) was fabricated via electrospinning and carbonized through a thermal process. Before carbonization, oxidative stabilization should be applied, and the oxidation mechanism also plays an important role during carbonization. Thus, the understanding of the oxidation mechanism is an essential part of the production of CNF. The oxidation process of polyacrylonitrile was studied and nanofiber webs containing graphene oxide (GO) are obtained to improve the electrochemical properties of CNF. Structural and morphological characterizations of the webs are carried out by using attenuated total reflectance Fourier transform infrared spectroscopy and Raman spectroscopy, scanning electron microscopy, atomic force microscopy and transmission electron microscopy. Mechanical tests are performed with a dynamic mechanical analyzer, and thermal studies are conducted by using thermogravimetric analysis. Electrochemical impedance spectroscopy, and cyclic voltammetry are used to investigate capacitive behavior of the products. The proposed equivalent circuit model was consistent with charge-transfer processes taking place at interior pores filled with electrolyte.

## Introduction

Carbon nanofibers are of great interest because of their chemical similarity to fullerenes and carbon nanotubes. Carbon nanofibers (CNF) have promising electrochemical and mechanical properties and a potential for a variety of applications; such as supercapacitor applications, battery applications, and catalyst support materials. Polyacrylonitrile (PAN) is one of the well-known precursor for obtaining carbon nanofibers that have a diameter ranging between nanometers and micrometers and exhibit a high surface area and a high electrical conductivity.

Also, nanofibers can be used with polymeric structures to generate composite materials to improve the electrochemical properties of polymeric structures [1–3]. Nanofiber-reinforced polymeric structures present improved mechanical properties because of the interaction between nanofibers and the matrix material [4]. CNF can be used as reinforcing material inside the polymer composites thanks to their enhanced mechanical and physical properties [5–7]. The manufacturing of CNF/polymer composites is challenging and the manufacturing processes need to be improved to obtain high-performance composite structures [8].

Oxidative stabilization is a crucial heat-treatment process to produce carbon fibers from PAN fibers. PAN chains start to cross-link during this process and the newly composed polymeric structure can endure the rigors of high-temperature processing [9–11]. Oxidative stabilization is crucial to prevent melting or fusion of the fibers. Also, it minimizes volatilization of elemental carbon in the following carbonization step and maximizes the final carbon yield. Chemistry and mechanisms of complex oxidative stabilization reactions for PAN were reported [12]. Oxidative stabilization reactions mainly consist of dehydrogenations and cyclizations, i.e., cyclization of nitrile groups (C≡N) and crosslinking of chain molecules in the form of –C=N–C=N–. Moreover, this stabilization process depends on pyrolysis temperature, heating rate, tension of the fiber, total stabilization time and dwell time, air flow rate and pre-stabilization treatment [13]. Carbonization is the next step in the process. The carbonization processes can be divided into low-temperature and high-temperature carbonization, and graphitization above 2000 °C [14–16]. Carbonization should be conducted under nitrogen environment to prevent burning [17–19]. During the carbonization process, the elimination of other elements (N_2_, O_2_, H_2_) and structural impurities is accelerated and the carbon concentration inside the structure is simultaneously increased.

The most common co-monomers of acrylonitrile in the acrylonitrile copoymers are: vinyl acetate, itaconic acid, methyl metacrylate and acrylic acid [18–20]. Co-monomers are mainly used is to improve the processability of acrylonitrile and to decrease the cyclization temperature [21–22]. For instance, the glass-transition temperature (*T*_g_) of PAN homopolymer is reduced by the addition of a co-monomer to form P(AN-*co*-AA), enhancing cyclization reactions and the formation of thermally stable aromatic ladder polymer chains [18]. Acidic co-monomers (itaconic acid and acrylic acid) improve the hydrophilicity of the PAN precursor but also catalyze the cyclization of nitrile groups during the stabilization process by forming a ladder structure. In our previous studies, copolymers of AN have been synthesized by free radical polymerization, and electrospun nanofibers were obtained with different AN co-polymers as carbon nanofiber precursors [13,18].

Graphene has several desirable features, such as high surface area, high aspect ratio and other properties comparable to those of carbon nanotubes. Thus, graphene attracts attention in science as a new class of material for polymer-based composites [23]. Graphene oxide has been synthesized from graphite with strong acids and oxidants [24–25]. The oxidation level can be adjusted by modifying reaction conditions and systems, and the type of precursor. Moreover, oxygen functional groups increase wettability and capacitance, but not all of the surface oxygen groups have the same effect. For enhancing the capacitance of a supercapacitor, an active electrode material with oxygen functional groups is necessary [24]. Furthermore, the PAN cyclization temperature can be decreased in the presence of graphene oxide. The functional groups of graphene oxide initiate the PAN cyclization at lower temperature via ionic mechanisms. In addition, the performance of an electrochemical capacitor prepared from carbon nanotubes/carbon nanofiber (CNT/CNF) composites is influenced by the oxidation level. Increasing the O/C ratio improves the capacitance of CNT/CNF composites. According to literature, a flexible and free standing composite paper comprising carbon nanofibers and graphene shows a higher specific capacitance than pure carbon nanofibers. Thus, the CNF/graphene combination can be a good candidate for a high-performance flexible capacitor applications [26].

In this paper, graphene oxide was used as an additive to increase the capacitance of oxidized PAN-based nanofibers. Further, GO addition was studied to improve electrochemical properties of CNF webs.

## Experimental

### Materials

Polyacrylonitrile (PAN, *M*_w_ 150,000 g/mol) was purchased from Sigma-Aldrich and was used as received. Dimethylformamide (DMF; Sigma-Aldrich), sulfuric acid (H_2_SO_4_, 98%; Sigma-Aldrich), acetonitrile (ACN; Sigma) were chosen as solvents and were used without any further purification. Graphene oxide (GO, purity 99%) was purchased from Grafen Chemical Industries and used as received. The properties of the few-layered GO are: GO consists of a few layers (1–10 layers) and the average thickness of the layer is smaller than 4 nm. The specific surface area of GO is larger than 550 m^2^/g. GO consists of 68.44 atom % C, 30.92 atom % O and 0.63 atom % S.

For electrospinning, PAN dissolved in DMF and spinning solution was prepared. The solution was fed into a 2 mL syringe and under high voltage (around 15 kV) DMF evaporated and nanofiber formation was achieved on the collector. Those nanofibers stacked and formed a web. Different collectors were used to fabricate PAN-based nanofiber webs via electrospinning. Before the electrospinning process, GO was also added to the PAN/DMF solution to obtain PAN/GO nanofibers. After electrospinning of the PAN/GO nanofibers, the PAN/GO samples underwent the same heat treatment (oxidation and carbonization) as the PAN nanofibers. Rotating and fixed collectors were used to vary the samples and investigate physical and chemical changes.

Electrospinning solutions were prepared at different PAN/DMF ratios. Electrospinning parameters (e.g., viscosity, voltage, feeding ratio) effect the nanofiber diameter and homogeneity. Lower viscosity helps to produce finer nanofibers, and an increased polymer weight percentage results in higher viscosities. Thus it is one of the significant parameters for electrospinning [27].

In this study, graphene oxide was used as an additive to increase the capacitance of oxidized PAN-based nanofibers. Thus, the nanofibers were produced via electrospinning using a mixture of PAN (10% w/v) and a given amount of GO (at different weight-to-volume percentages) in DMF. The solutions were poured into a 2 mL syringe and delivered at a constant flow rate of 1.0 mL/h (New era, NE-300) to a needle with a blunt tip connected to a high-voltage power supply (Gamma high voltage research) producing a voltage of 15 kV. Aligned nanofibers were deposited on the rotating drum collector at 21.50 Hz rotating frequency at a distance of 15 cm. After producing the nanofibers, oxidative stabilization was performed at 250 °C for 3 h in air atmosphere and carbonization was performed at 900 °C for 1 h under nitrogen atmosphere.

### Characterization

Attenuated total reflectance Fourier transform infrared spectroscopy (ATR-FTIR) and Raman spectroscopy were used to record the characteristic peaks of the oxidized and carbonized nanofibers. Mechanical properties of nanofiber webs were characterized by using a dynamic mechanical analyzer (DMA) (TA Q800 Dynamic Mechanical Analyser).

Thermal behavior of nanofiber webs was examined with thermogravimetric analysis (TGA, Q 50 from TA instruments). The structure of the nanofiber webs was characterized by attenuated total reflectance Fourier transform infrared spectroscopy (ATR-FTIR) (Perkin Elmer, Spectrum One, with a Universal ATR attachment with a diamond and ZnSe crystal). The microstructure of the carbonized nanofiber webs was investigated by Raman spectroscopy (DXR Raman spectrometer, Thermo Scientific, at 532 nm). The sample morphologies were characterized by scanning electron microscopy (Gemini Leo Supra 35 VP) and samples were coated with thin gold film using a sputter coater to prevent the accumulation of charge on their surface.

Electrochemical performances of nanofibers were analyzed by using cyclic voltammetry (CV) and electrochemical impedance spectroscopy (EIS). Electrochemical measurements were performed by using potentiostat 2263 Electrochemical Analyser (Princeton Applied Research, Tennessee, USA). EIS data were simulated with the electrical equivalent circuit by ZSimpWin V.3.10 analysis program (Princeton Applied Research, Tennessee, USA). The surface topography of the fibers was observed by atomic force microscopy (AFM) with Nanosurf Easy-Scan2^TM^ software. AFM analyses were performed with a non-contact mode by using NCLR-10 model Al-coating silicon tips with 7 μm thickness, 225 μm length, 38 μm width, 190 kHz resonance frequency and 48 N/m force constant. Surface morphology of the nanofibers was observed with scanning electron microscopy (SEM) at Namık Kemal University and transmission electron microscopy (TEM). Fiber diameters were measured within electron micrographs from a population between forty and fifty nanofibers taken from each sample and then the average values were calculated by ImageJ software.

## Results and Discussion

### Oxidative stabilization of PAN nanofibers

Oxidative stabilization is a complex process and should be applied to the webs before carbonization. The mechanism plays an important role in the carbonization. Therefore, a detailed understanding of the mechanism of oxidation has an important part in the success of the production of CNF.

**Figure 1:**
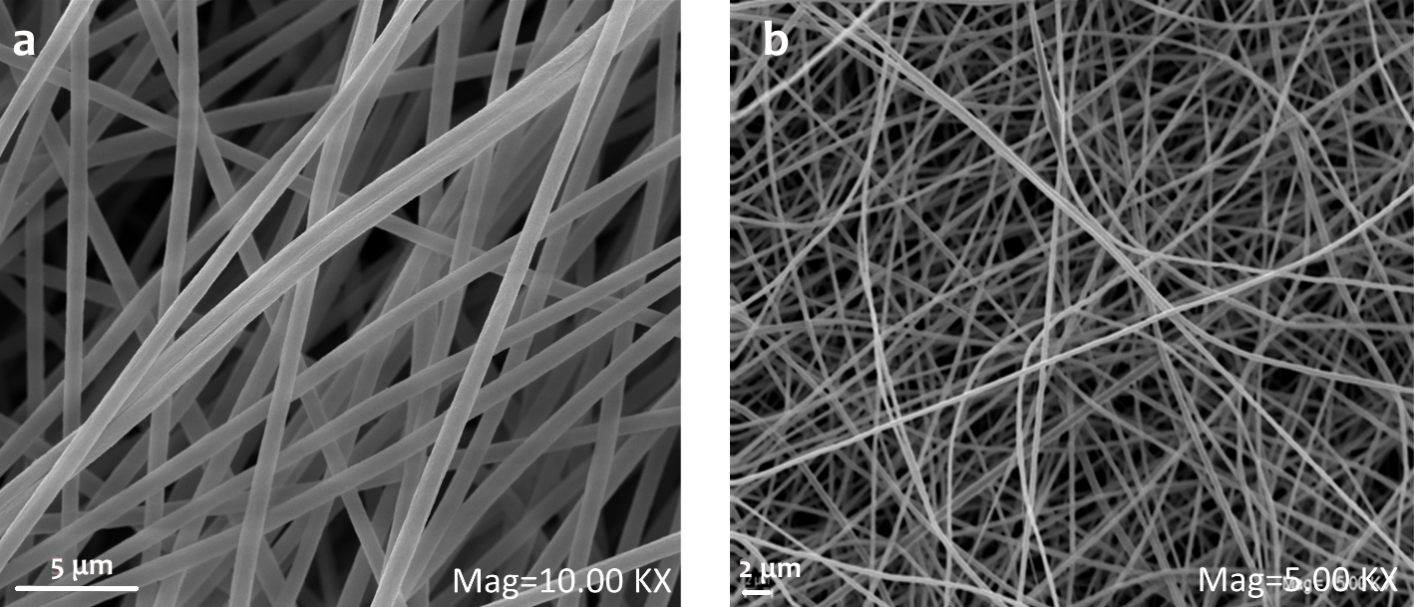
a) Web of aligned PAN nanofibers produced with rotating collector and b) web of PAN nanofibers produced with fixed collector.

**Figure 2:**
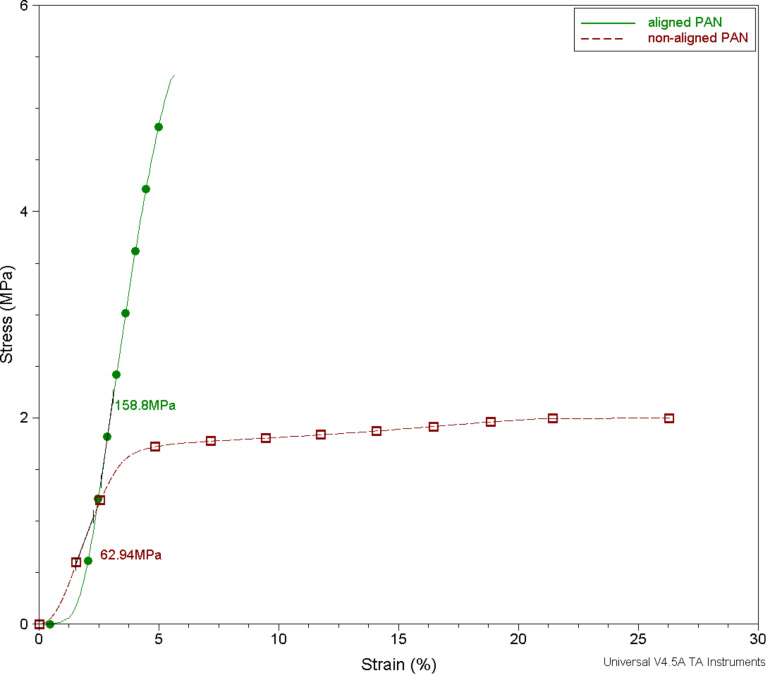
Stress–strain plots of webs of aligned and non-aligned PAN nanofibers.

Nanofiber webs are produced with different collectors to achieve fiber alignment. The results for webs of aligned and non-aligned nanofibers are compared. A rotating collector that produces aligned nanofiber webs reduces the nanofiber diameter as shown in SEM images in Figure 1. Non-aligned PAN nanofibers diameter are in the range of 371.6 ± 36 nm; whereas the aligned PAN nanofibers diameter are decreased to 330.8 ± 27 nm. The stress–strain curve obtained by DMA shows that fiber alignment increases the mechanical properties of the web. A directional orientation of the fibers definitely and expectedly has the effect of increasing modulus and reducing the strain to break [18,28–29]. Aligned nanofibers has a greater modulus than non-aligned ones [18,28,30–32]. Also, our previous work [33] exhaustively explains the effects of rotating collector and fixed collector. Rotational movement helps to orient the nanofibers and obtain thinner fibers compared to the fixed collector. Webs of aligned nanofibers present superior mechanical properties in terms of modulus. Figure 2 shows stress–strain plots of aligned and non-aligned PAN nanofibers. According to the plots, the elastic modulus of a PAN-nanofiber web increases with fiber orientation from 63 MPa to 159 MPa. Thus, rotating collectors were chosen to obtain nanofibers with better mechanical and morphological properties.

### ATR-FTIR spectroscopy results

**Figure 3:**
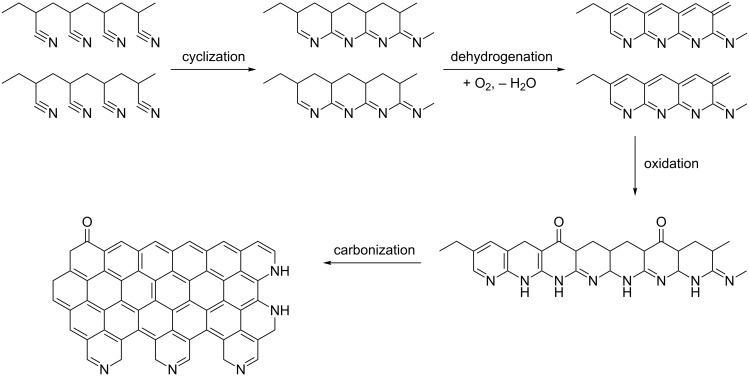
Schematic description of carbonization process starting from polyacrylonitrile.

**Figure 4:**
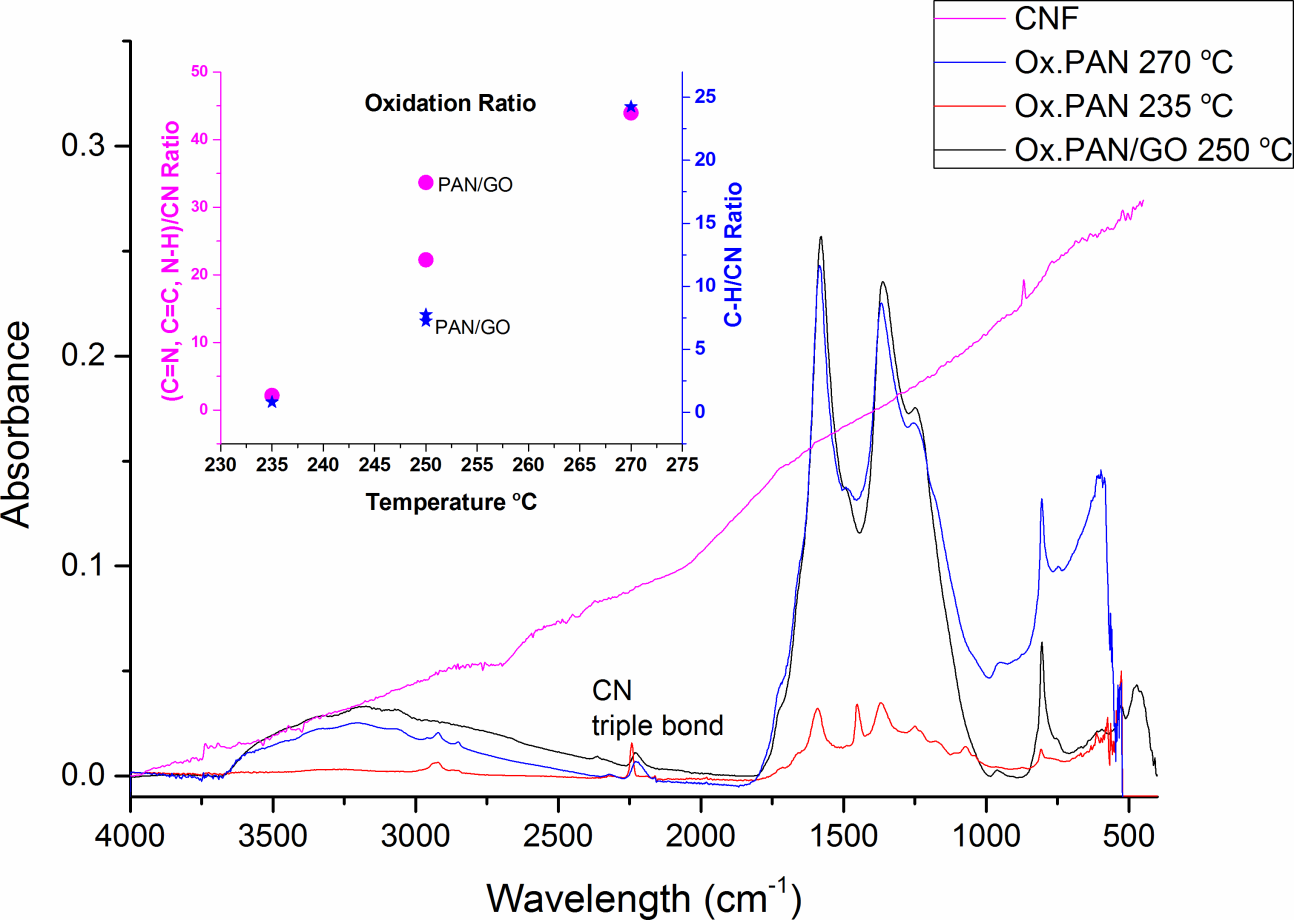
ATR-FTIR results of oxidized webs with GO and of the carbon nanofiber web. The inset represents the oxidation ratio of the webs as a function of the temperature according to absorbance ratios of (C=N, C=C, N–H)/C≡N and the newly occurred =C−H/C≡N ratio. (C=N, C=C, N–H) represents the mixture of the corresponding absorbances.

The oxidation process including conversions of C≡N bonds to C=N and also dehydrogenation leading to aromatic and supramolecular structures was studied [34]. Structural changes during the oxidation process can be tentatively expressed as in Figure 3, and the oxidation route was explained through cyclization and dehydrogenation reactions. Peaks around 2243 cm^−1^ represent the absorption of C≡N triple bond [17,22,35]. Those around 1590 cm^−1^ can be assigned to a combined effect of C=N, C=C, N–H groups [12,15,17,35–36], and the broad peak at around 3000 cm^−1^ is connected to C–H bonds [37–38]. ATR-FTIR results are given in Figure 4. The oxidation temperature is too low to eliminate all C≡N triple bonds. This means that cyclization reactions cannot be completed. A schematic description is given in Figure 3. However, the intensity of the C≡N triple bonds is decreased after oxidation [12,36,39]. A weight loss is not observed during the cyclization process, contrary to dehydrogenation [37]. During the dehydrogenation a new peak appears at around 800 cm^−1^ because of the formation of =C−H bonds [38,40–41]. In the presence of oxygen =C−H groups were created during the aromatization by the removal of H atoms in the form of H_2_O [38]. Also, an increased temperature increases the intensity of the =C−H peak.

The oxidation ratio can be calculated from the absorbance ratio obtained from ATR-FTIR results [12,39–40]. The inset in Figure 4 represents the oxidation ratio as a function of the oxidation temperature by evaluating the ratio between the mixed signals of C=N, C=C, N–H groups and the signal of C≡N triple bonds. At oxidation temperatures of 250 °C and 270 °C, the oxidation ratios are quite close contrary to that of the oxidation at 235 °C. During the oxidation process, C≡N triple bonds are damaged and C=N double bonds are created. Thus the ratios of these peaks from ATR-FTIR can help to calculate the oxidation ratio. GO-containing samples are marked in the inset Figure 4. At 250 °C, the addition of GO to the PAN nanofiber web causes a deviation in the oxidation ratio values compared to pure PAN. GO acts via ionic mechanism in the oxidation step and improves the conversion of C≡N bonds to C=N, C=C and N–H [35,42–43].

During the stabilization process, the cyclization of the nitrile groups and cross-linking of the chain molecules is followed by dehydrogenation [38]. This reaction promotes the creation of a ladder structure from the linear molecule [36,38,44]. Ladder-structure polymers are thermally more stable than linear polymers, because the structure prevents them from melting at higher temperatures [38,45]. Weight loss starts at around 100 °C with the removal of moisture and continues with increasing duration and temperature. However, it is not much significant for the oxidation process [12,18,46]. Weight loss as a function of temperature and time was recorded with TGA. There is a region in which there is no weight loss, and this region can be explained by cyclization reactions [37]. Both TGA curves (in N_2_ and in O_2_ atmosphere) exhibit the same trend. However, the region with no weight loss is shifted in N_2_ atmosphere because of N_2_ suppresses the reactions compared to O_2_, according to TGA measurements, the PAN polymer stays stable up to ca. 300 °C. This stable phase can be explained by cyclization reactions [37]. Above this temperature, weight loss begins to increase because of the dehydrogenation reactions [37–38]. In O_2_ atmosphere, weight loss starts above 100 °C, after a stable cyclization phase, dehydrogenation in O_2_ atmosphere is observed between 100 and 140 °C. In N_2_ atmosphere this temperature shifts to 300–400 °C. The reaction propagation is faster under O_2_ atmosphere compared to N_2_.

**Figure 5:**
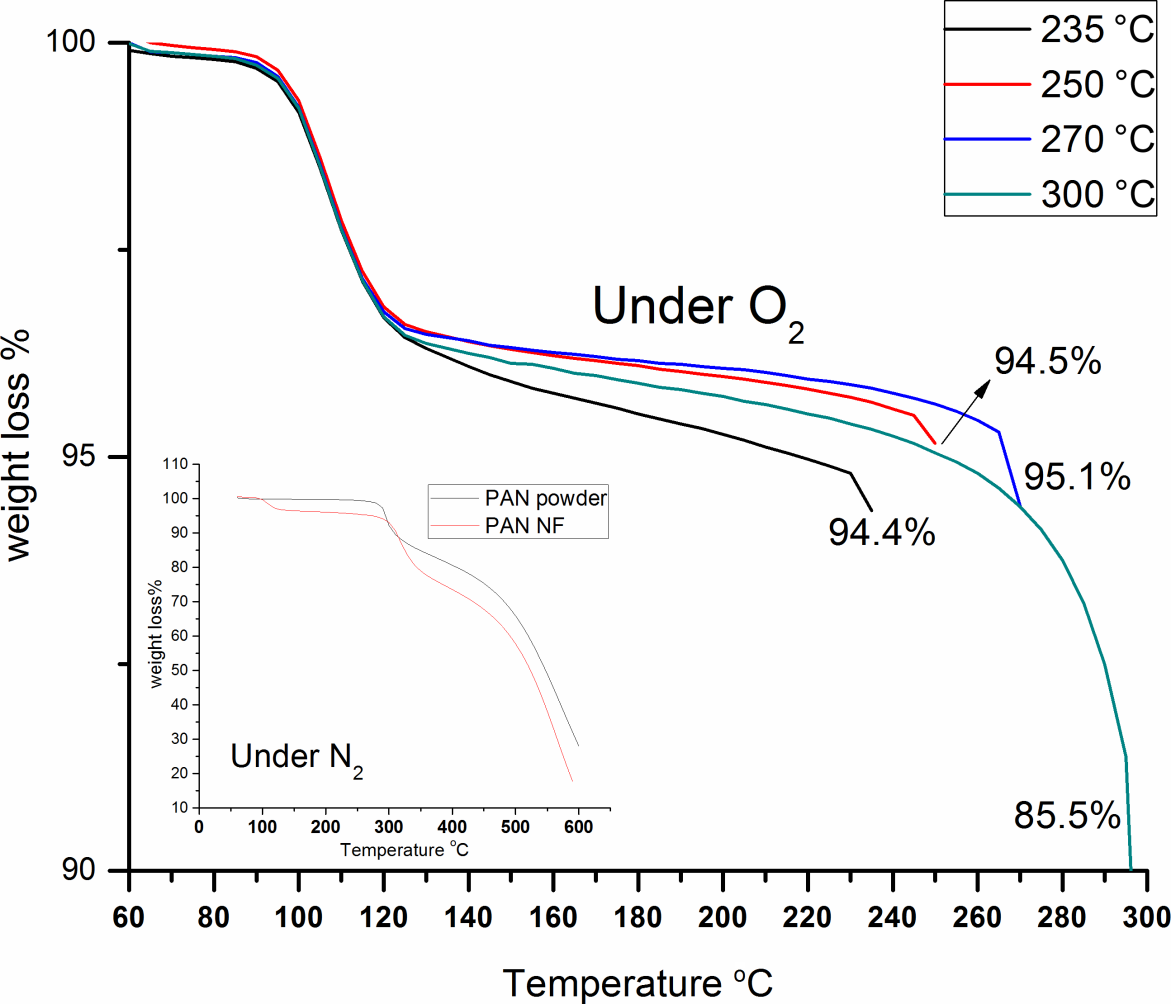
TGA curves representing the experimental conditions of oxidation for 300 min of different oxidation temperatures for webs of aligned PAN nanofibers (under air). The inset represents the TGA of PAN polymer under inert (nitrogen) atmosphere.

The same conditions as in the oxidation procedure were applied during TGA. A 5 °C/min ramp was applied till the samples reached the desired oxidation temperature (235, 250, 270 and 300 °C). After the samples reached the oxidation temperature TGA was carried out for further 300 minutes. It can be seen from the TGA curves that the 300 °C/300 min oxidation process shows the highest weight loss. For the TGA measurement of nanofiber webs in oxygen environment the curves are similar to those of the PAN polymer. However, the temperature ranges are shifted because of the presence of oxygen. In the presence of reactive atmospheres, such as air or oxygen, the oxidation process is faster at lower temperatures [47]. At temperatures above 100 °C weight loss was recorded. A sudden reduction of weight was recorded during dehydrogenation reactions in which hydrogen and oxygen formed H_2_O, which was released from the structure [37–38]. Figure 5 shows that at for the oxidation temperature of 300 °C a weight loss of around 14.5% is observed after 300 min. During the 300 min of oxidation, the weight loss varies between 4.9% and 14.5%, for increased temperatures (235, 250 and 270 °C). For 300 °C the weight loss is recorded as 14.5%. The energy applied to the sample depends on temperature and duration. Together they promote bond breakage, thus the weight loss of the samples increases with temperature. Also, using a co-polymer instead of a homopolymer can strengthen the fiber structure and lead to a higher heat stability [13,18]. A dramatic weight loss (around 45%) is recorded during the low-temperature carbonization process with increasing elimination of other elements (N,H,O) [38,48].

### Electrochemical impedance measurements of oxidized PAN nanofibers

Electrochemical properties of oxidized PAN nanofibers were analyzed by using electrochemical impedance spectroscopy (EIS). EIS measurements were performed in 0.5 M H_2_SO_4_ electrolyte in the frequency range of 100 mHz to 100 kHz at open circuit potential with an AC perturbation of 10 mV. A standard three-electrode cell was used to study the electrochemical performances of PAN nanofibers which were stabilized at 250 °C for 1 h in air. Oxidized PAN nanofiber mats were used as free standing working electrodes, a platinum wire was used as counter electrode, and a silver wire was used as pseudo-reference electrode. EIS data were simulated with electrical equivalent circuit by using the ZSimpWin V.3.10 analysis program.

**Figure 6:**
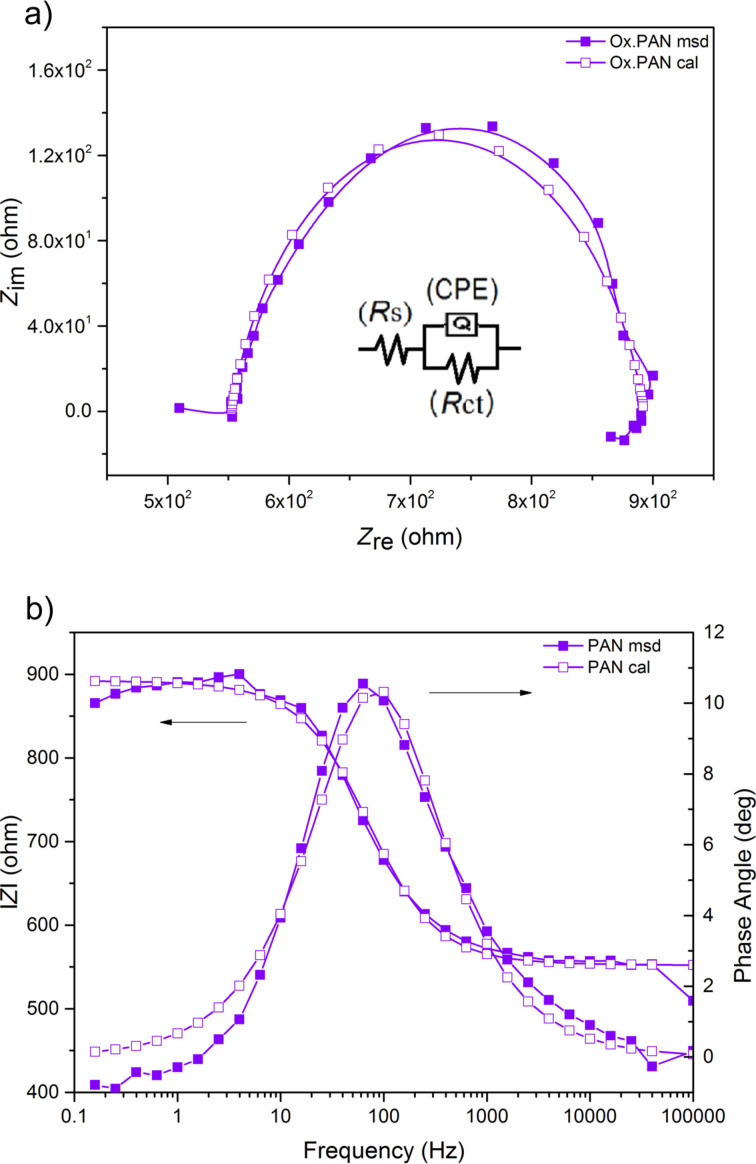
a) Nyquist plots of webs of oxidized PAN nanofibers. Inset: Randles circuit model. b) Bode magnitude and Bode phase plots of PAN nanofiber webs.

Experimental and calculated measurements were fitted by equivalent circuit modelling. EIS plots with measured and calculated data are shown in Figure 6. An excellent agreement between experimental results and simulation was found with χ^2^ ≈ 5·10^−4^ (χ^2^ is function defined as the sum of the squares of the residuals). *R*_s_ is the ohmic resistance of the solution, *R*_ct_ represents the charge-transfer resistance between nanofiber electrodes and electrolyte interface and *Q*_dl_ (constant phase element (CPE)) is the double-layer CPE, a frequency-dependent element.

The Nyquist plot in Figure 6a consists of a semicircle related to the electron-transfer process. The charge-transfer resistance (*R*_ct_) can be calculated from measuring the diameter of the semicircle. According to the Bode phase plot in Figure 6b, the phase angle of the sample was 10° around 80 Hz. In the Bode magnitude plot, the absolute values of impedance are plotted as a function of the frequency. The impedance values between low-frequency region and high-frequency region do not change drastically compared to the GO-containing PAN nanofibers (see below in Figure 14). Addition of GO to PAN nanofibers changes the homogeneity of the electrode. Thus, the penetration of electrolyte ions penetration varies with frequency.

The values of *R*_s_, *R*_ct_ and *Q*_dl_ were determined as 552 Ω, 340 Ω and 2·10^−2^ µS·s*^n^* according to the Randles circuit model for non-ideal electrodes described as *R*_s_(*Q*_dl_*R*_ct_) in short hand. The CPE (*Q*_dl_) can also be attributed to the double-layer capacitance (*C*_dl_) in the non-homogeneous systems [49]. Double-layer capacitance occurs at the electrode/electrolyte interface of materials with especially high surface area. The electrical charge is stored based on the separation of charged species in an electrolytic double layer across the interface of electrode/solution. This capacitance value is proportional to the surface area of the electrode and inversely proportional to the thickness of the double layer [50].

The impedance of the non-ideal electrode is defined by

[1]
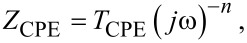


where *j* is the imaginary unit √−1, ω is the angular frequency, and *T*_CPE_ and *n* are frequency-independent experimental constants; *T*_CPE_ relates to the size, thickness, and materials properties, while *n* relates to the degree of energy dissipation and measures the arc depression, which is frequency-independent. Moreover, *n* is a parameter describing the deviation from an ideal capacitor and arises from the slope of the log *Z* versus log *f* plot. The values for *n* vary from 0 to 1, and *n* = 1 describes an ideal capacitor, while *n* = 0 describes the behavior of a resistor. The *n* value of oxidized PAN was equal to 0.83.

### Oxidative stabilization of PAN/GO nanofibers and CNF

**Figure 7:**
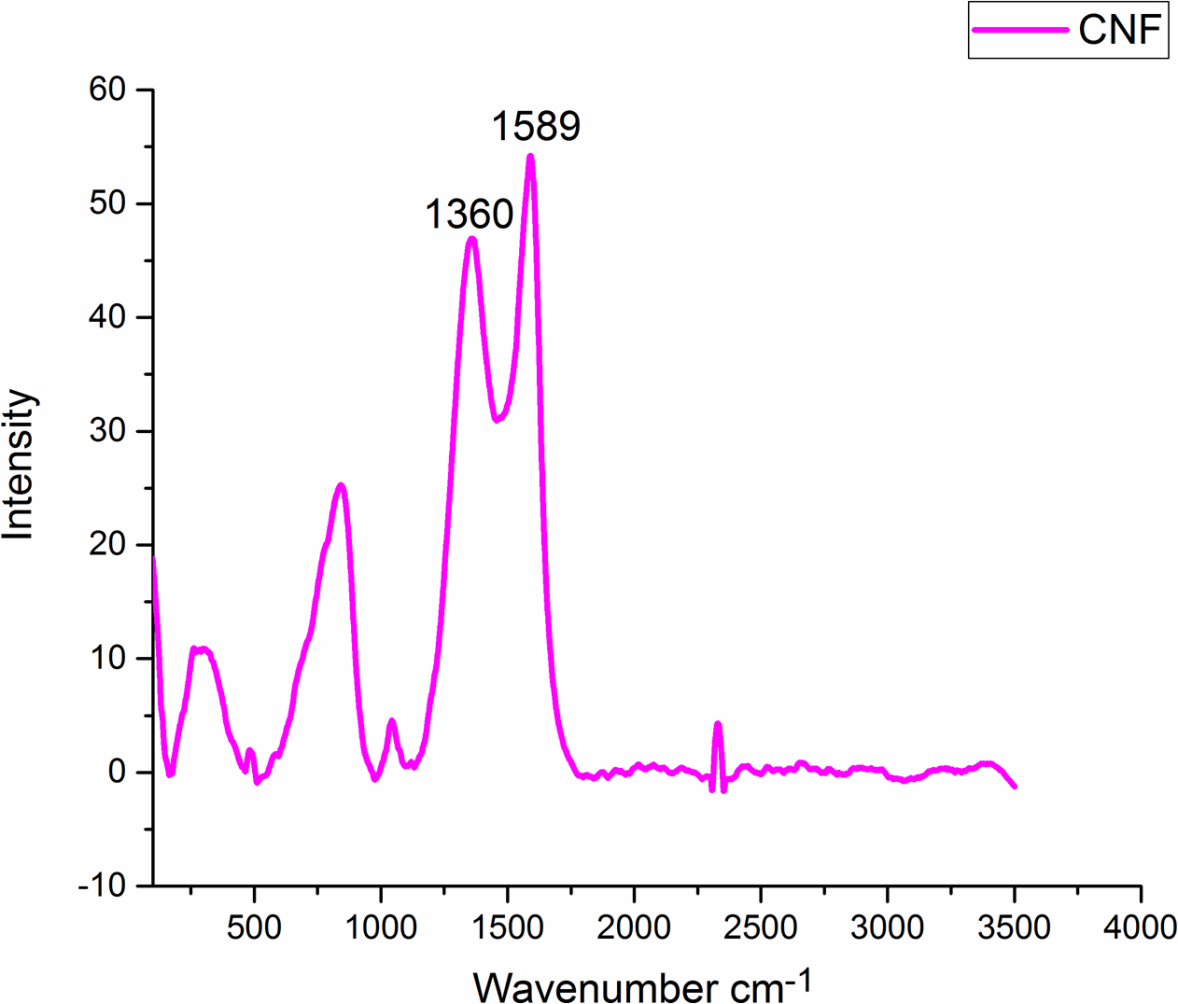
Raman spectrum of carbon nanofiber webs.

The Raman spectroscopic measurements show characteristic peaks of carbon materials, namely D band and G band at around 1360 cm^−1^ and 1580–1600 cm^−1^, respectively [51–52] (Figure 7). Oxidation and carbonization contributed to the conversion of PAN fibers into a graphitic form via fraction of disordered sp^2^-hybridized C–C bonds [53]. The ratio of the D and G bands provides an information about the crystallinity of the carbonaceous material [52,54]. The G band (1590 cm^−1^) represents ordered graphitic crystallites [52], while the D band around 1350 cm^−1^ is related to disordered turbostratic structures [55]. The measured intensity ratio between D band and G band (*R* = *I*_D_/*I*_G_) indicates structurally ordered graphite crystallites [30,54]. The *R* value of CNF is around 0.9. A lower *R* value means a more crystalline material with higher conductivity [56]. Position and intensity of D and G band demonstrate the electronic structure and electron–phonon interactions of the material [51].

### ATR-FTIR spectroscopy of oxidized PAN/GO nanofibers

**Figure 8:**
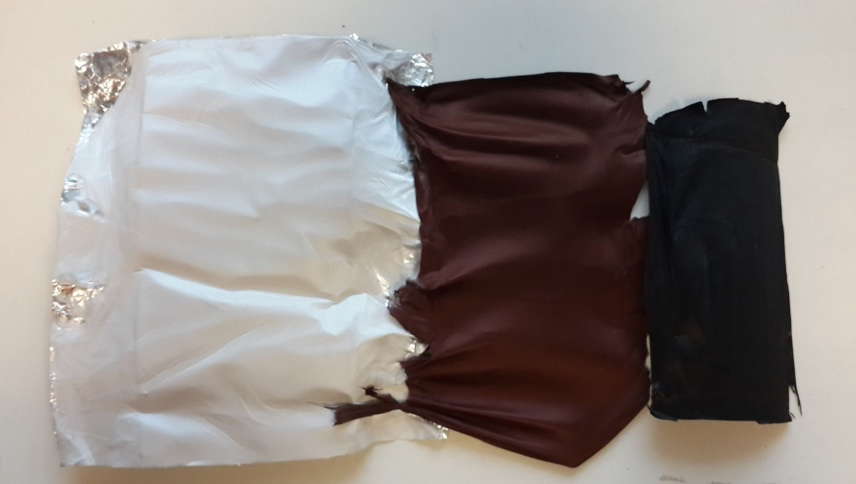
GO-containing PAN-based electrospun, oxidized and carbonized nanofibers.

The ATR-FTIR results show a broad OH stretching peak of GO around 3300 cm^−1^ [57] and the C–H vibrations of the CH, CH_2_ and CH_3_ structures of oxidized polyacrylonitrile around 2920 cm^−1^ [38,40]. Through the carbonization process most of the bonds are damaged and eliminated. The ladder structure of carbon atoms becomes more dominant and it is not always possible to follow further structural changes of carbonaceous materials with FTIR. Also, ATR-FTIR results of carbonized nanofibers (Figure 4) are not clear not only because of the changing bond structure of PAN but also because of the black color of the carbon nanofiber webs. A photo of GO-containing PAN-based electrospun, oxidized and carbonized nanofibers are shown in Figure 8. The colors of the nanofibers change from white to brown after oxidation and then from brown to black after carbonization.

### Morphologic studies

**Figure 9:**
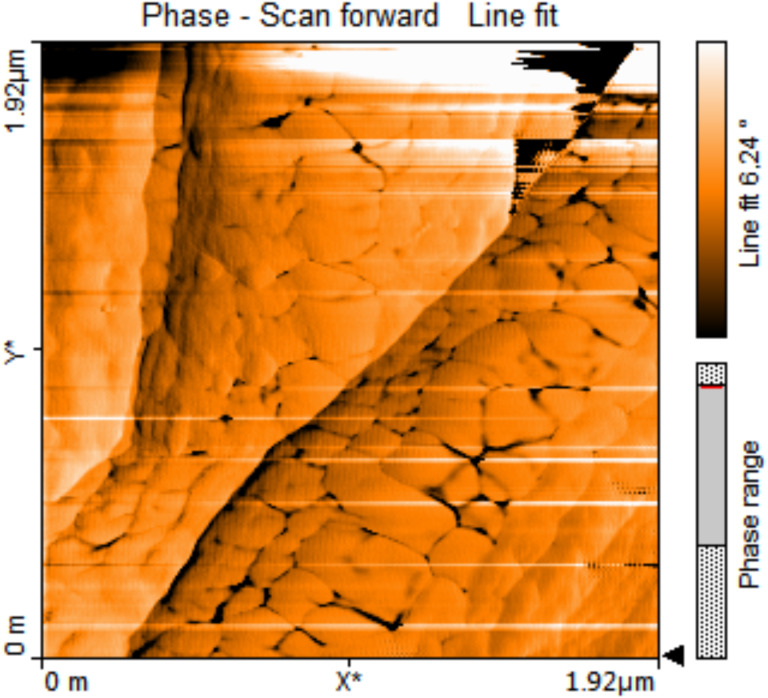
AFM image of GO-containing oxidized PAN nanofiber webs.

**Figure 10:**
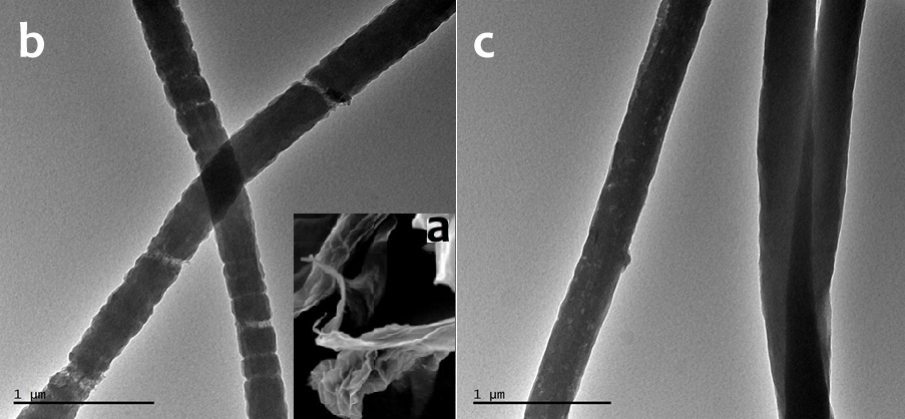
a) SEM image of GO; b) TEM images of GO-containing PAN nanofibers; c) carbon nanofibers.

**Figure 11:**
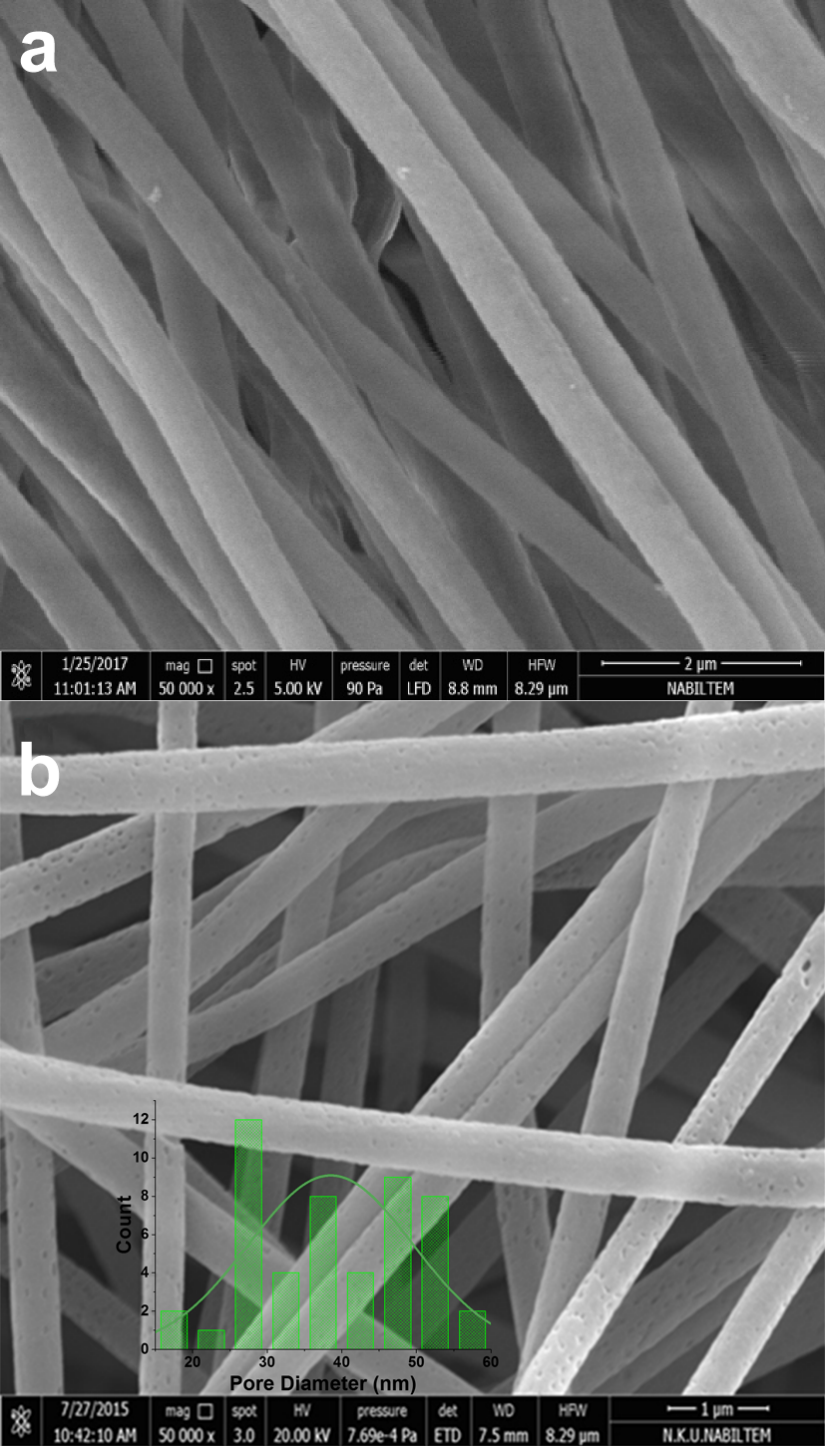
SEM images of (a) oxidized PAN nanofiber webs and (b) GO-containing oxidized PAN nanofiber webs with pore distribution chart.

The surface of the nanofibers is not smooth and has pores, which can be related to graphene oxide content. This can be seen very clearly from the AFM, SEM and TEM images in Figure 9, Figure 10 and Figure 11. AFM was performed to observe the topography of nanofibers. Oxidized PAN nanofibers formed with GO nanosheets can be seen in AFM image (Figure 9). The nanofibers have rough surfaces with flaky shapes attributed to GO. The morphology of GO is also shown in Figure 10a. Layers of GO can be seen in the SEM image. Also, some layer edges of GO and the interspaces of the layers can be observed in the SEM image. GO-containing electrospun nanofibers are seen in Figure 10b,c. GO nanosheets that are formed with PAN nanofibers are observed on the structure in Figure 10b. A rough surface with a kind of joints is presented in the image. Distance between two nodes in the structure is around 50 nm calculated by ImageJ Software.

The morphology of PAN nanofibers with smooth surfaces is presented in Figure 1 and Figure 11a for comparison with GO-containing PAN nanofibers. When GO is included into PAN nanofibers rough surface can be seen. Furthermore, the porous structure of carbon nanofibers with GO is shown in Figure 10b,c. Figure 11b represents the porous surface of oxidized PAN/GO nanofibers and a pore distribution chart was added on the SEM image. It can be seen from Figure 11 that addition of GO makes the nanofiber surface porous and these pores are well distributed on the fibers. The morphological property of the porous carbon electrodes such as the surface and pore size distributions are the factor that influences the double-layer capacitance. Therefore, the pore size distribution of porous carbons also affect the performance of carbon-based electrochemical capacitors [58].

According to SEM images (Figure 11b) pore size on the nanofibers were measured as 38.5 ± 11 nm. All morphologic characterizations prove the porous structure of GO containing nanofibers.

In supercapacitors that use nanoporous electrodes to store large amounts of charge, ions penetrate into the pores of the electrode. Raymundo-Piñero et al. considered that an adequate pore size is more important than a high surface area and reported optimum pore sizes as 0.7 nm and 0.8 nm in aqueous and organic media, respectively [59]. Graphene oxide shows a high specific capacitance because of layered graphene sheets [24].

### Electrochemical impedance studies of PAN and GO-containing PAN-based nanofibers

A standard three-electrode cell was used to study electrochemical performances of nanofibers by using cyclic voltammetry (CV) and electrochemical impedance spectroscopy (EIS). Carbonized nanofibers were used as free standing electrodes whereas oxidized nanofibers were deposited on fluorine-doped tin oxide (FTO) glass to use as working electrodes. EIS analysis were investigated in 0.1 M NaClO_4_/ACN electrolyte in a frequency range of 10 mHz to 100 kHz at open circuit potential with an AC perturbation of 10 mV. The samples of oxidized nanofibers are designated as Ox.PAN, Ox.PAN/GO(1) and Ox.PAN/GO(2) indicating concentration of 0, 1.25 and 2.5% graphene oxide relative to PAN, respectively.

**Figure 12:**
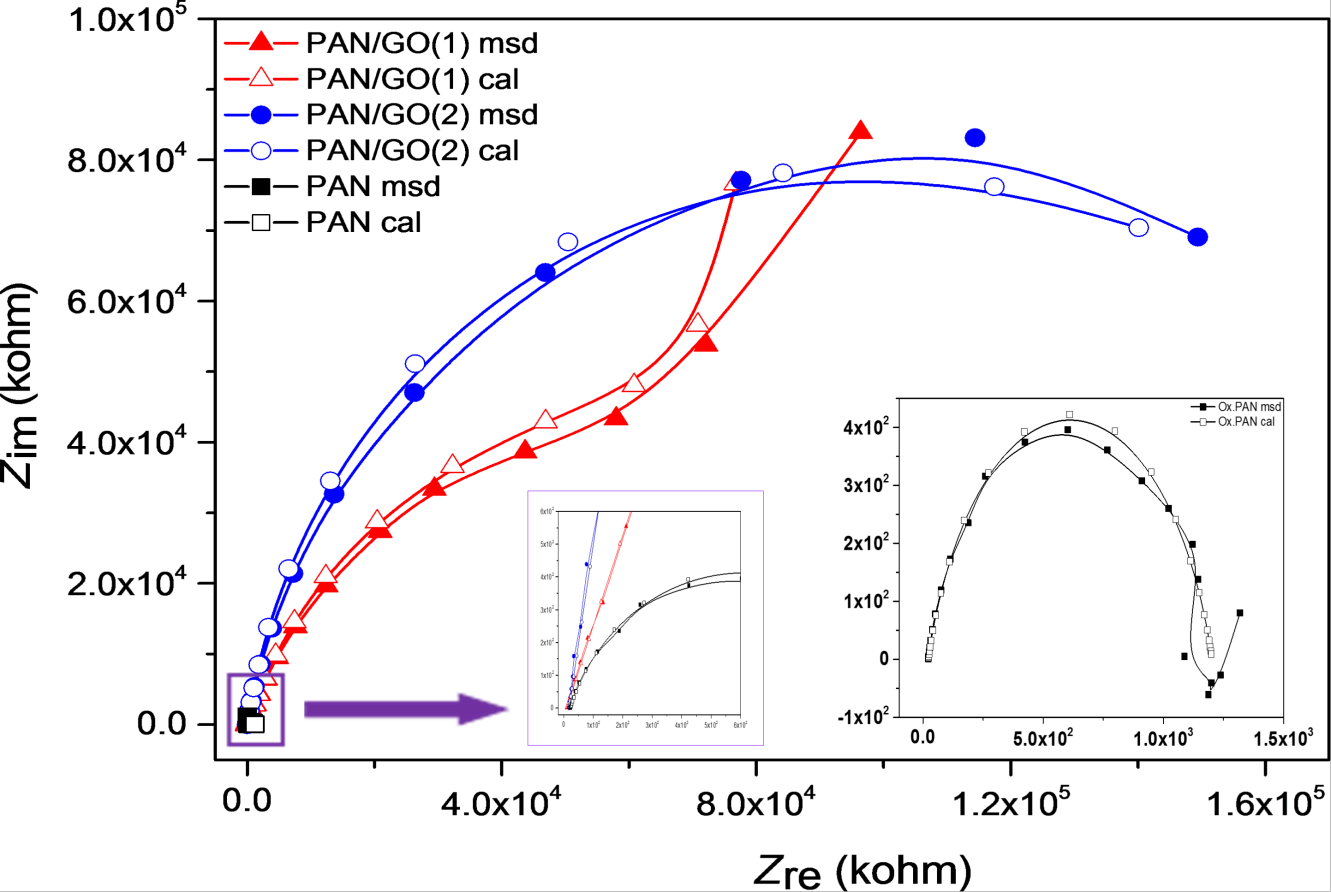
Nyquist plots of oxidized PAN and GO-containing oxidized PAN nanofiber webs (inset: Nyquist plots of oxidized nanofiber webs at high frequencies and Ox.PAN nanofiber webs up to 100 kHz).

Nyquist plots in Figure 12 represent a semicircle in the high to medium frequency range. The inclined line corresponding to diffusion processes at low frequencies region appears only in PAN/GO(1). The charge-transfer resistances (*R*_ct_) were evaluated by using equivalent circuit modelling. *R*_ct_ is attributed to the pore size of the electrodes. The values of *R*_ct_ of Ox.PAN, Ox.PAN/GO(1) and Ox.PAN/GO(2) were equal to 1180 kΩ, 119700 kΩ and 182800 kΩ, respectively, *R*_ct_ increases with GO content.

**Figure 13:**
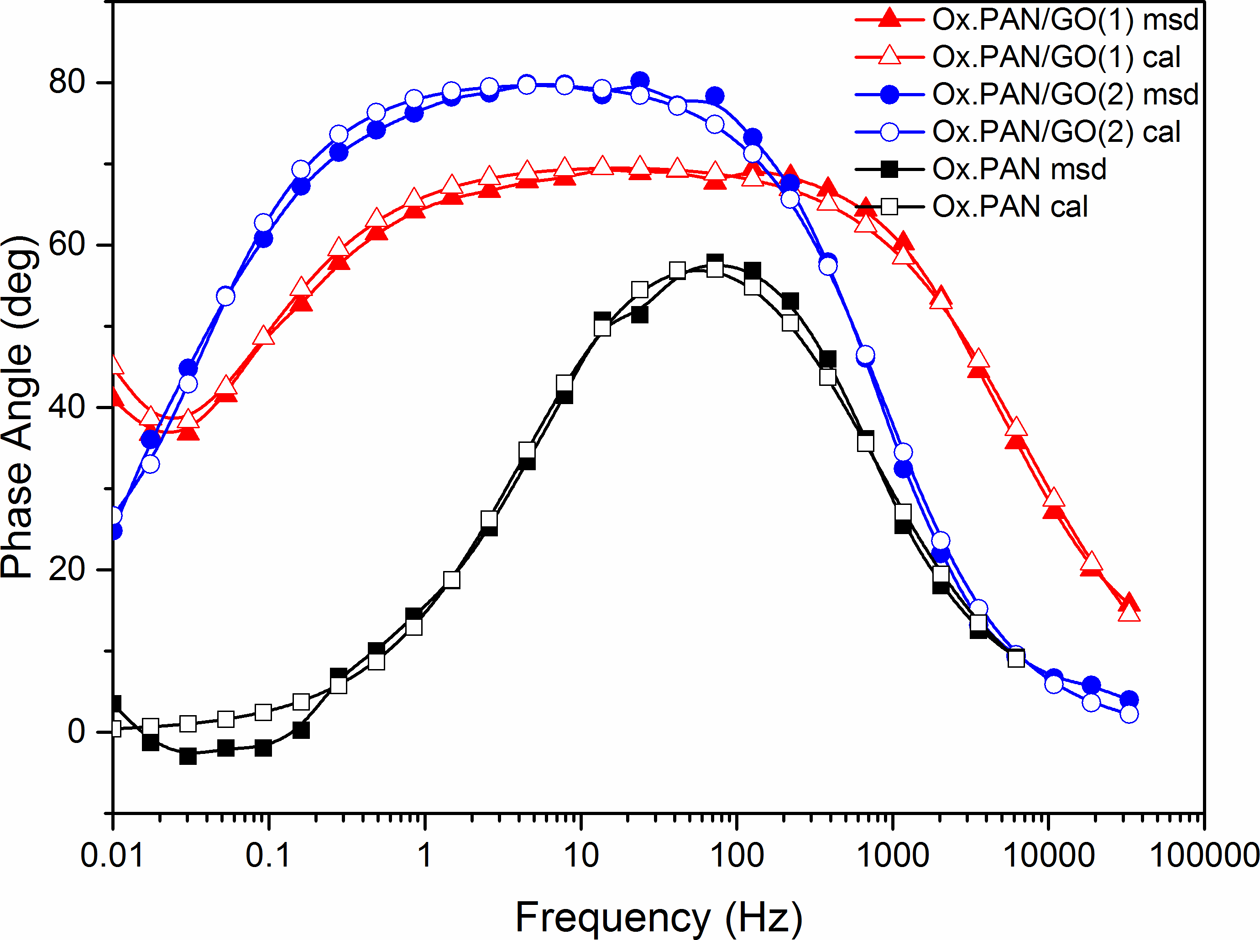
Bode phase plots of oxidized PAN nanofiber webs and GO-containing oxidized PAN nanofiber webs.

According to the Bode phase plots, the sample of Ox.PAN/GO(1) and Ox.PAN/GO(2) show similar properties while Ox.PAN behaves differently (Figure 13). After adding GO to the nanofibers the phase angle increases linearly and exhibits larger plateau regions. This indicates the capacitive behavior.

**Figure 14:**
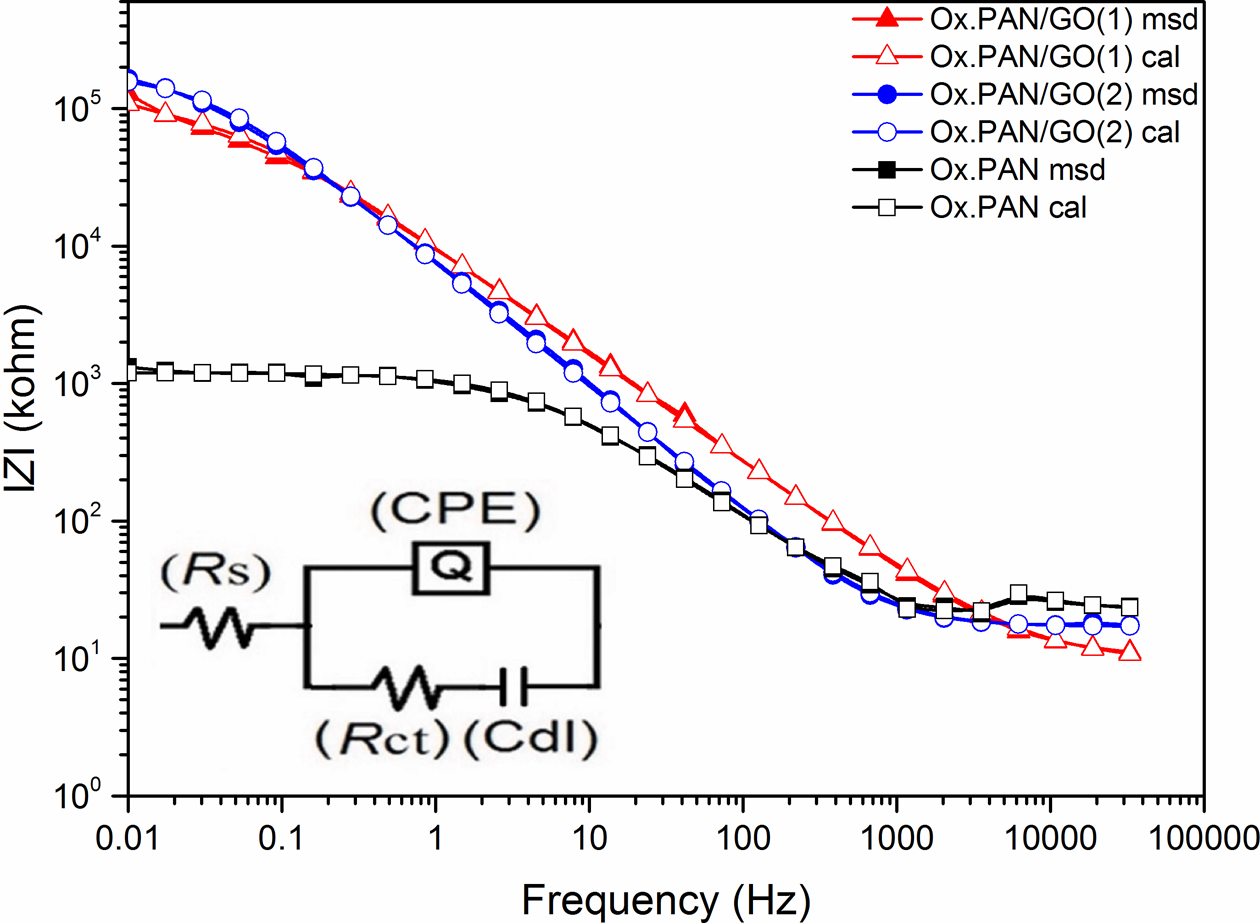
Bode magnitude plots of oxidized PAN nanofiber webs and GO-containing oxidized PAN nanofiber webs (inset shows the electrochemical equivalent circuit).

The Bode magnitude plots exhibit two different shapes for high and low frequencies (Figure 14). At high frequencies, the impedance values of Ox.PAN and Ox.PAN/GO nanofibers do not change significantly and this is attributed to the disability of the electrolyte ions to penetrate into the electrode. The solution resistance (*R*_s_) of the electrochemical system changes very slightly, which can be seen in Table 1. On the other hand, the impedance of Ox.PAN/GO nanofibers is very high due to the penetration of ions into the electrode surfaces at low frequencies [24].

**Table 1:** Fitting values for the equivalent circuit elements by simulation of the impedance spectra of oxidized nanofibers.

sample	*R*_s_ (kΩ)	*Q*_el_ (CPE) (µS·s*^n^*)	*n*	*C*_dl_ (µF)	*R*_ct_ (kΩ)	χ^2^ (10^−3^)

Ox.PAN^a^	21.80	0.060	0.79	—	1180	5.54
Ox.PAN/GO(1)	9.55	0.024	0.78	0.190	119700	4.72
Ox.PAN/GO(2)	17.05	0.025	0.90	0.600	182800	3.99

^a^An *R*_s_(*Q*_el_*R*_ct_) equivalent circuit model has shown a better correlation with this sample.

The parameters of the simulated equivalent circuit models obtained from the Nyquist and Bode phase plots are given in Table 1. Fitting with equivalent circuit modelling exhibited a good correlation between the calculated and experimental values with χ^2^ values around 10^−3^. The result shows two different models. *R*_s_(*Q*_el_*R*_ct_) circuit modeling was compatible with Ox.PAN, while a *R*_s_(*Q*_el_(*R*_ct_*C*_dl_)) circuit modelling was chosen for Ox.PAN/GO(1) and Ox.PAN/GO(2).

*R*_s_ corresponds to the solution resistance, *R*_ct_ corrresponds to the charge-transfer resistance of electrode surface and solution interface, and *Q*_el_ corrresponds to the combined capacitance of nanofibers and FTO glass electrode. *R*_ct_ and *C*_dl_ change linearly with the amount of GO and the values of *n* of Ox.PAN and Ox.PAN/GO(1) are is very similar (Table 1). After increasing the GO content in the nanofibers, the value of *n* increases and exhibits nearly ideal capacitive behavior for Ox.PAN/GO(2). The *C*_dl_ value of Ox.PAN/GO(2) is 3.16 times higher than that of Ox.PAN/GO(1). A CPE is generally used in heterogeneous systems associated with non-ideal capacitive behavior resulting from electrode roughness, inhomogeneous conductivity, or even diffusion [60]. CPE is also related to the composition of the nanofibers. The proposed model is consistent with charge-transfer processes taking place at interior pores filled with electrolyte.

**Figure 15:**
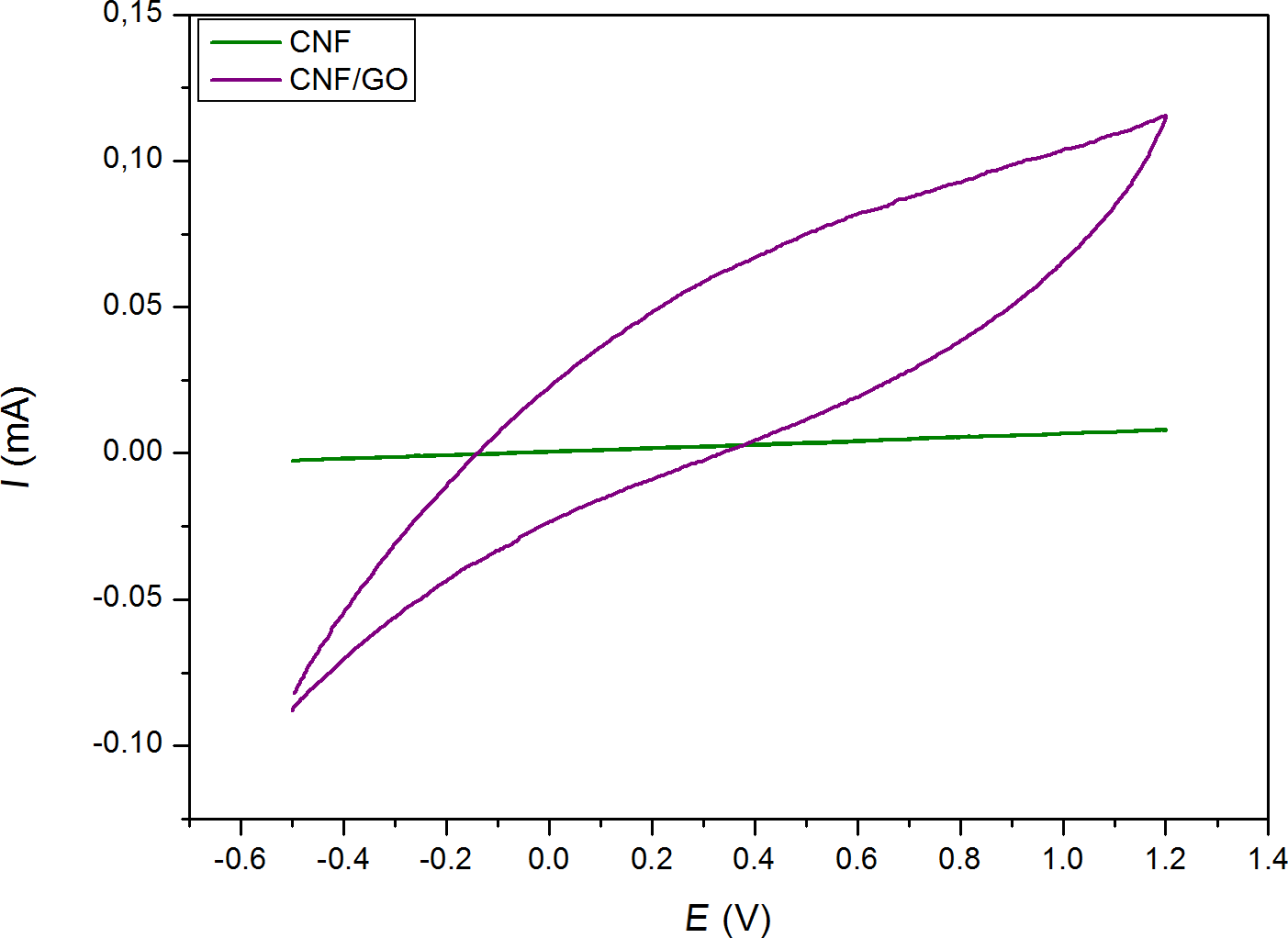
Cyclic voltammograms of carbon nanofibers and GO-containing carbon nanofiber webs at scan rate of 50 mV·s^−1^ (PAN-based nanofibers with and without GO, first oxidized then carbonized).

Heat treatment was applied to Ox.PAN and Ox.PAN/GO nanofibers to produce carbon nanofibers (CNF) and GO-containing carbon nanofibers (CNF/GO). CNF and CNF/GO, which include very small amount of graphene oxide (1.25% relative to PAN) were used as free standing working electrodes during CV. Figure 15 shows the CV of CNF and CNF/GO electrodes at a scan rate of 50 mV·s^−1^ between −0.5 V and 1.2 V in 0.1 M NaClO_4_/ACN electrolyte. It can be seen that CNF/GO electrode exhibits a larger CV area than the CNF electrode, indicating a higher specific capacitance compared to CNF. Adding GO increases the O/C ratio, which could result in an enhanced capacitive behavior of the carbon nanofibers.

## Conclusion

In this paper, CNF webs and GO-containing CNF webs were successfully fabricated. Nanofiber webs were fabricated via electrospinning. Nanofiber alignment was achieved with a rotating collector, which also had the definite and expected effect of increasing modulus and reducing the strain to break of the webs. Different oxidation temperatures were studied and 250 °C was selected as optimum temperature for this study. Increased the oxidation temperature increases the oxidation level of the sample. However, thermal oxidation between 200 and 300 °C was not enough to eliminate all C≡N triple bonds. GO-containing oxidized nanofibers have a rough surface. Nanopores of around 38.5 ± 11 nm pore size on the nanofiber surface can help to store large amounts of charge. GO addition into PAN makes a significant change on the EIS results, i.e., the capacitive behavior increases with the increase in the *C*_dl_ value of GO-containing oxidized nanofibers. The *C*_dl_ value of Ox.PAN/GO(2) is the highest as being 0.600 µF. Individual layered sheets of GO with high surface area are supposedly exposed to the electrolyte, which can result in the increase of the double layer capacitance. GO functional groups enhance the capacitance performance of CNF webs. As a result, CNF/GO can be a potential candidate for capacitive applications.
